# Effects of Diet Induced Weight Reduction on Cartilage Pathology and Inflammatory Mediators in the Joint Tissues

**DOI:** 10.3389/fmed.2021.628843

**Published:** 2021-03-22

**Authors:** Antonia RuJia Sun, Xiaoxin Wu, Ross Crawford, Hongxing Li, Lin Mei, Yong Luo, Yin Xiao, Xinzhan Mao, Indira Prasadam

**Affiliations:** ^1^School of Mechanical, Medical, and Process Engineering, Institute of Health and Biomedical Innovation, Queensland University of Technology, Brisbane, QLD, Australia; ^2^Center for Translational Medicine Research and Development, Shenzhen Institutes of Advanced Technology, Chinese Academy of Sciences, Shenzhen, China; ^3^Department of Orthopaedic Surgery, The Second Xiangya Hospital, Central South University, Changsha, China; ^4^Orthopedic Department, The Prince Charles Hospital, Brisbane, QLD, Australia; ^5^Australia–China Centre for Tissue Engineering and Regenerative Medicine, Queensland University of Technology, Brisbane, QLD, Australia

**Keywords:** osteoarthritis, diet induced obesity, infrapatellar fat pad, synovium, inflammation, cartilage

## Abstract

Obesogenic diets contribute to the pathology of osteoarthritis (OA) by altering systemic and local metabolic inflammation. Yet, it remains unclear how quickly and reproducibly the body responds to weight loss strategies and improve OA. In this study we tested whether switching obese diet to a normal chow diet can mitigate the detrimental effects of inflammatory pathways that contribute to OA pathology. Male C57BL/6 mice were first fed with obesogenic diet (high fat diet) and switched to normal chow diet (obese diet → normal diet) or continued obese diet or normal diet throughout the experiment. A mouse model of OA was induced by surgical destabilization of the medial meniscus (DMM) model into the knee joint. Outcome measures included changes in metabolic factors such as glucose, insulin, lipid, and serum cytokines levels. Inflammation in synovial biopsies was scored and inflammation was determined using FACs sorted macrophages. Cartilage degeneration was monitored using histopathology. Our results indicate, dietary switching (obese diet → normal diet) reduced body weight and restored metabolic parameters and showed less synovial tissue inflammation. Systemic blood concentrations of pro-inflammatory cytokines IL-1α, IL-6, IL-12p40, and IL-17 were decreased, and anti-inflammatory cytokines IL-4 and IL-13 were increased in dietary switch group compared to mice that were fed with obesogenic diet continuously. Although obese diet worsens the cartilage degeneration in DMM OA model, weight loss induced by dietary switch does not promote the histopathological changes of OA during this study period. Collectively, these data demonstrate that switching obesogenic diet to normal improved metabolic syndrome symptoms and can modulate both systemic and synovium inflammation levels.

## Introduction

Obesity and its associated metabolic disorders are an important contributor to the progression of OA (1–5). It is well-accepted that obesity is mainly caused by an imbalance between energy intake and expenditure that promotes storage of nutrient oversupply in white adipose tissue (6). Although the underlying mechanisms that link OA with obesity remain to be elucidated, the state of chronic low-grade inflammation in obesity, which is caused by an increased accumulation of macrophages in the adipose tissue, has been proposed (7). As the links between obesogenic diet, inflammatory responses and OA have been established, it is critical to investigate whether these changes are reversible by weight loss following nutritional changes, as obesity may be the only risk factor that is modifiable. In mice and monkeys, a 20–40% reduction of dietary caloric intake can reduce body weight, extending lifespan, improving metabolic disturbances and decreasing the systemic inflammatory status through changes in inflammatory gene expression, reduction of oxidative stress, decreased metabolism and increased capacity of DNA repair (8–10). Substantial evidence also supported that weight loss of 20% in patients with obesity and OA by gastric surgery led to an improvement in pain and physical function (11). Moreover, there was an inverse dose-response relationship between weight loss and progression of cartilage abnormalities in obese patients with knee OA (12, 13). Although weight loss has been extensively studied regarding its short-term benefits on major symptoms in obese patients with chronic diseases including OA (13, 14), the effect of weight loss on the progression of morphologic abnormalities of the knee joints remains a key area of ongoing research.

We have previously reported that consumption of a high-fat, high-carbohydrate diet promoted systemic and local synovial inflammation and contributed to development of OA (5). We have further reported that dietary intake of long-chain saturated fatty acids was associated with the development and acceleration of OA (15, 16). This study used diet-induced obese rodents with OA to investigate whether switching to normal chow effect cartilage pathology and synovial inflammation. The aim of this study is to test if dietary-induced reversal of OA would be concurrent with reversal of the systemic and local synovial inflammatory state, metabolic syndrome characteristics and cartilage degradation.

## Methods and Materials

### Animals

Animal experimental protocols were approved by the Institutional Animal Care and Use Committees and Institutional Biosafety Committees of Central-South University (CSU; 2013-05), China. Female C57BL/6 mice were purchased from the Animal Center of Central-South University (Changsha, Hunan, China). Mice had free access to food and water during the experimental protocols. At the start of the experimental protocols, mice were 6 weeks old.

### Dietary Interventions

**Figure 1:**
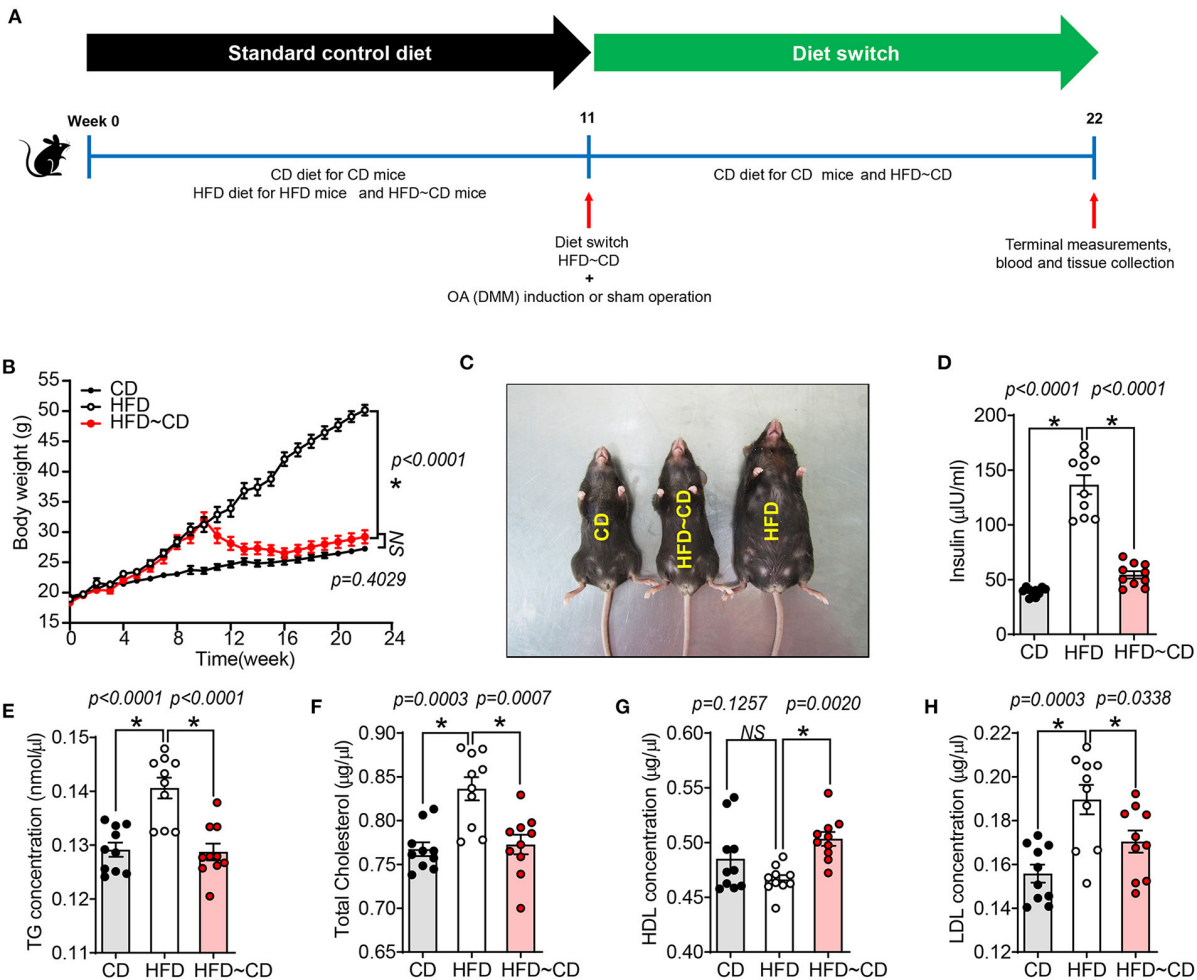
High fat diet -induced obesity and altered metabolic parameters are reversed by diet-initiated weight loss. **(A)** Schematic diagram showing the experimental procedure. Six-week-old female C57BL/6 J mice were fed a CD or HFD for 11 weeks where half of the HFD-mice were switched to or continued a CD or HFD for additional 11 weeks, respectively. CD-fed mice were maintained on the same diet. After 11 weeks of feeding, mice either underwent surgery for destabilization of the medial meniscus (DMM) to induce knee OA in the left knee joint or a sham operation that did not dissect the medial meniscal ligament. CD, control diet; HFD, high fat diet; HFD~CD, high fat diet switched to control diet. **(B)** Body weight of CD, HFD and HFD~CD mice were monitored over 22 weeks. **(C)** Dorsal view of the mice showing the changes caused by the diet after 22 weeks. **(D–H)** Effect of diets on metabolic parameters in mice. Measurement of serum insulin **(D)**, total triglyceride **(E)**, total cholesterol **(F)**, HDL **(G)**, LDL **(H)**. Graphs represent mean ± SD (*N* = 10 per group). *Significant differences between results in different group (i.e., *p* < 0.05).

A total of 60 C57BL/6 mice were included in this study. Mice were randomly divided into six groups. Group 1: Control diet (CD), Group 2: High fat diet (HFD), Group 3: Dietary switch group (HFD diet → CD diet), Group 4: Sham surgery + fed with CD, Group 5: OA surgery + fed with HFD diet, Group 6: OA surgery + dietary switch HFD diet → CD diet). C57BL/6 mice were initially placed on a standard chow diet or control diet composed of 10 kcal% Fat (D12450B, Research Diets) and an obesogenic diet or HFD composed of 60 kcal% fat (D12492, Research Diets, Chengdu, China). After 11 weeks of feeding, some mice underwent surgery for destabilization of the medial meniscus (DMM) to induce knee OA in the right knee joint or a sham operation that did not dissect the medial meniscal ligament as described previously (17). Mice were randomized for the remainder of the study to either continue the CD diet; continue the HFD; or HFD switch to CD (Figure 1A). Mice were group-housed in a temperature-controlled room on 12-h light/dark cycles with routine veterinary assessment. Body weight and food consumption measures were recorded weekly. After euthanasia, blood was withdrawn by cardiac puncture, and the serum was collected for cytokine analysis. Serum was used for biochemical analyses according to previous studies (15).

### Biochemical and Metabolic Parameters

Blood samples collected during the terminal experiments were analyzed to determine the concentrations of insulin and lipids. Serum insulin was measured using enzyme-linked immunosorbent assay (ELISA) kit following the manufacturer's instructions (Abcam, Changsha, Hunan, China). Serum total cholesterol (TC), high-density lipoprotein cholesterol (HDL-c) and low-density lipoprotein cholesterol (LDL-c) were using a Cholesterol Assay Kit (Abcam, Changsha, Hunan, China). The concentration of serum triglyceride was determined using a Triglyceride Quantification Kit (BioVision, Dakewe Biotech, Beijing, China). Serum of mice were obtained by centrifuging at 1,500 × g for 10 min, and levels of cytokines were measured with a Bio-Plex™ Mouse Cytokine 23-Plex Panel (#M60009RDPD, BioRad, Life Science, China) by using a BioRad Bio-Plex 200 System according to the manufacturer's instruction. These measured cytokines were as follows: IL-1α, IL-1β, IL-2, IL-3, IL-4, IL-5, IL-6, IL-9, IL-10, IL-12 p (40), IL-12 p (70), IL-13, IL-17A, eotaxin, G-CSF, GM-CSF, IFN-γ, KC, MCP-1, MIP-1α, MIP-1β, RANTES, and TNFα.

### Isolation of Synoviocytes and Sorting Macrophages

Isolation of synovium was performed according to published methods (18–20). Briefly, immediately after euthanasia, the synovial tissues around the knee joints were collected and pooled from 3 animals per group. The synovium was minced and digested in a 1 mg/ml collagenase type I (Sigma-Aldrich) for 1 h at 37°C and rinsed through a 70-μm filter (BD Biosciences). The isolated synoviocytes were suspended in phosphate-buffered saline (PBS) containing 20 μg/ml of antibody cocktail. Brilliant Violet 510- and phycoerythrin-conjugated antibodies against mouse CD45.2 and F4/80 were obtained from Biolegend (Chaoyang, Beijing, China). For isotype control, Brilliant Violet 510- or phycoerythrin-conjugated non-specific mouse or rat IgG2a were substituted for the primary antibody, respectively. After incubating with antibody cocktails for 30 min at 4°C, the cells were washed with PBS and resuspended in PBS and macrophages were sorted using FACS and RNA was isolated from these sored cells.

### RNA Extraction, Reverse Transcription, and Gene Expression Profiling by Real-Time PCR

Immediately after tissue digestion, isolation of total RNA from sorted CD45.2+F4/80+ synovial macrophages were performed using TRIzol. RNA quantity and quality were assessed in a NanoDrop-100 spectrophotometer. cDNA synthesis from total RNA was performed using TaKaRa PrimeScript 1st strand cDNA Synthesis Kit according to the manufacturer's protocol. For quantification of gene expression by real-time PCR, SYBR Green detection chemistry was used on the Roche LightCycler 96 System. Quantitative measurements of all primers used in this study were determined using (2–ΔΔCt) method, and β-actin/GAPDH expression were used as the internal control (21–23).

### Histological Assessment of OA Development

The knee joints from seven mice per group were fixed overnight at 4% paraformaldehyde in 1X PBS, decalcified in 10% ethylenediaminetetraacetic acid (EDTA), embedded in paraffin and cut with a rotary microtome to generate 5-μm-thick sections. Sagittal sections were stained with safranin-O/fast green and haematoxylin & eosin to evaluate the disease severity by 2 observers under blinded conditions to dietary groups (24). The severity of OA was assessed in the medial compartment of the knee using the Mankin scoring system from 0 to 14 (Table 1) (21, 25). The degree of synovitis was scored using a 0–6 scoring system that measured the thickness of the synovial lining cell layer on a scale of 0–3 and cellular density in the synovial stroma on a scale of 0–3 as previously described (Table 2) (26). The sum of both parameters was used for analysis (26). Immunohistochemistry was performed (5, 15, 27) to determine the population of synovial macrophages and cartilage degradation products, respectively: anti-F4/80 (Abcam; Cat No: ab6640, Melbourne, VIC, Australia; dilution 1:100), anti-CD169 (Bioss Antibodies; Cat No: bs-10751R, Sapphire Bioscience, Redfern, NSW, Australia; dilution 1:100), anti-aggrecan NITEGE epitope (dilution 1:950) and anti-collagen DIPEN neoepitope (kind gift from Professor Amanda Fosang, Murdoch Children Research Institute, Melbourne, VIC, Australia; dilution 1:1240). The sections were incubated with corresponding secondary antibodies and the antibody complexes visualized using a diaminobenzidine (DAB) substrate and counterstained with Mayer's hematoxylin. Images were captured to perform histomorphometric measurements on the entire area of the knee synovial joint with a Leica SCN400 slide scanner (Leica Biosystems, Australia). Images were analyzed using Image J (National Institute of Health, Bethesda, BA, USA) for semi-quantitative data analysis. The positive cells within cartilage/synovium in each field (40 × objective lens) of observation were counted and normalized to the cell number per 100/total cells in each group. The number of positively stained cells in the medial compartment of the entire tibia above the tidemark per specimen in three sequential sections per mouse in each group. The total number of immuno-positive cells appearing within the synovium was estimated quantitatively in three sequential sections per animal in each group. Assessment of the histopathology and immunostaining was performed by two independent observers in a blind random manner.

**Table 1:** Mankin score grading system for osteoarthritic articular cartilage.

**Category**	**Subcategory**	**Score**
Structure	Normal	0
	Surface irregularities	1
	Pannus and surface irregularities	2
	Clefts to transitional zone	3
	Clefts to radical zone	4
	Clefts to calcified zone	5
	Complete disorganization	6
Cells	Normal	0
	Diffuse hypercellularity	1
	Cloning	2
	Hypocellularity	3
Safranin O staining	Normal	0
	Slight reduction	1
	Moderate reduction	2
	Severe reduction	3
	No dye noted	4
Tidemark integrity	Intact	0
	Crossed by blood vessels	1
Total		0–14

**Table 2:** Histopathological assessment of synovitis.

**Category**	**Subcategory**	**Score**
Enlargement of the synovial lining cell layer	Thickness 1–2 cells	0
	Thickness 2–4 cells	1
	Thickness 4–9 cells	2
	Thickness ≥ 10 cells	3
Cellular density in the synovial stroma	Normal cellularity	0
	slightly increased	1
	Moderately increased	2
	Greatly increased, pannus formation and rheumatoid-like granulomas might occur	3
Total		0–6

### Statistics

The minimum animal number and/or biological replicates for the primary outcome were determined by previous data (power = 0.8, *p* < 0.05, two-sided) (15, 16, 27). Briefly, *n* = 7/group for histology analysis; *n* = 10/group for metabolic parameters analysis; and *n* = 7/group for cytokine analysis. One-way ANOVA with Tukey's *post-hoc* analysis was used to determine whether parameters from the three groups of mice were significantly different using GraphPad Prism 7.0 for Windows (San Diego, CA, USA). Non-parametric data was evaluated using Friedman tests (Prism, GraphPad). Values are presented as mean ± SD for all variables unless indicated otherwise. Results were significantly different with *p* < 0.05.

## Results

### Changes in Obesogenic Diet-Induced Obesity Parameters Are Reversed by Weight Loss

HFD mice showed a significant increase in body weight compared to CD mice (50.17 ± 2.1 g vs. 27.26 ± 2.9 g) (Figures 1B,C). HFD-mice showed increased serum insulin concentrations compared to CD mice (Figure 1D). As shown in Figures 1E–H, total triglyceride (TG), cholesterol and low-density lipoprotein (LDL) concentrations in serum were markedly higher in HFD-mice, but no difference was observed in serum high-density lipoprotein (HDL) concentration compared to CD mice. Following weight loss of the HFD~CD group, serum insulin, TG, cholesterol, and LDL concentrations were decreased, and HDL concentrations were increased as compared to HFD-mice (Figures 1D–H). These parameters in the HFD~CD group returned to similar concentrations as in CD (Figures 1D–H).

### Effects of Diet Switching on Cartilage Pathology in Mouse Model of OA

**Figure 2:**
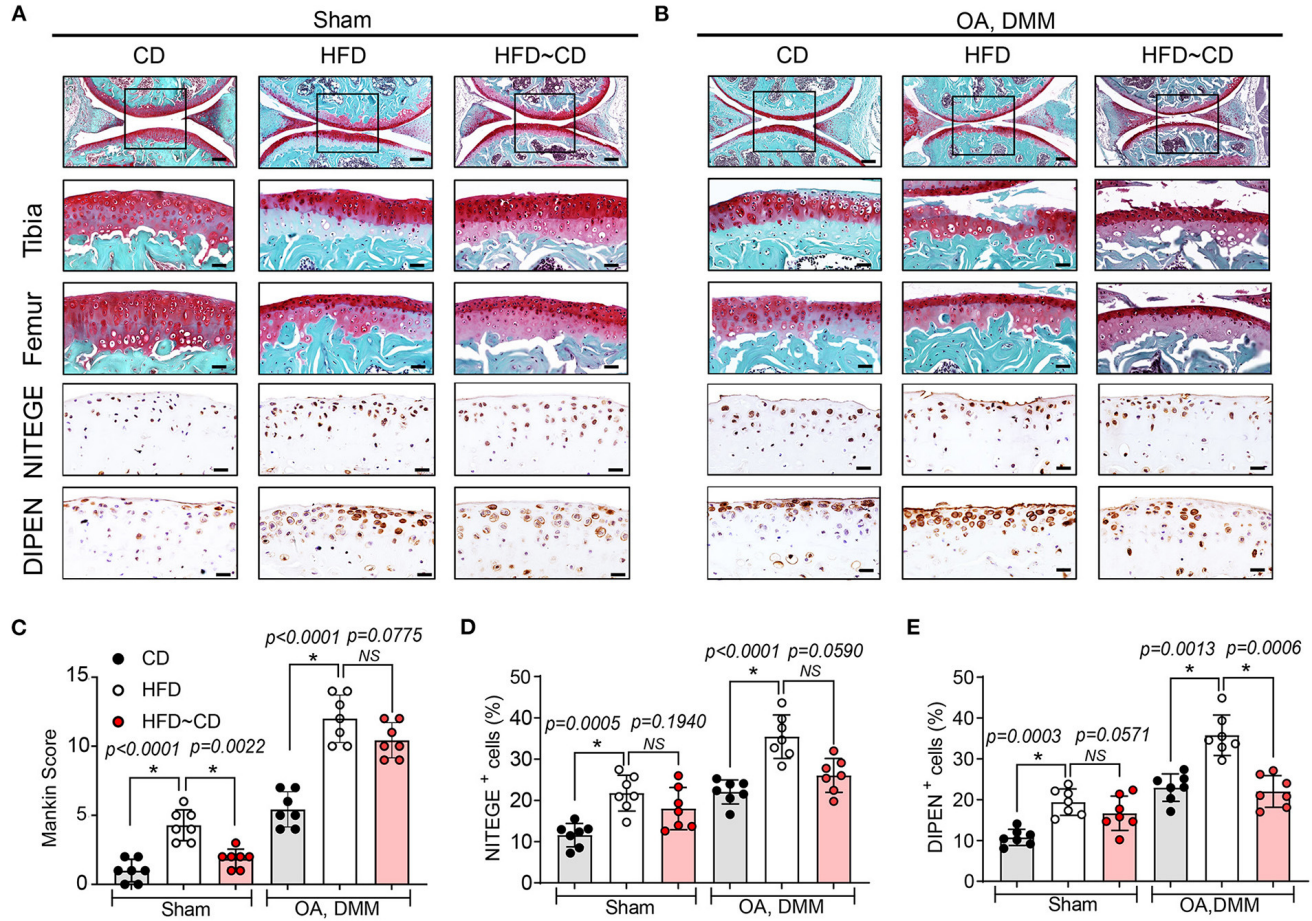
Effects of Diet switching on cartilage destruction in mouse model of OA. **(A)** Top panel: Representative Safranin O and fast green stained sagittal sections of sham knee regions in mice fed a CD, HFD, or HFD~CD. Scale bars, 100 μm. The inset boxes in upper panels are shown at higher resolution in lower panels. Scale bars, 20 μm. Bottom panel: Similar sections were stained with DIPEN and NITEGE. Scale bars, 20 μm. **(B)** Top panel: Representative Safranin O and fast green stained sagittal sections of OA-operated knee regions in mice fed a CD, HFD, or HFD~CD. Scale bars, 100 μm. The inset boxes in upper panels are shown at higher resolution in lower panels. Scale bars, 20 μm. Bottom panel: Consecutive sections were stained with DIPEN and NITEGE. Scale bars, 20 μm. *N* = 7 per group **(C)** Severity of articular cartilage degradation was graded using Mankin scoring system. **(D,E)** The percentage of DIPEN **(D)** and NITEGE **(E)**—positive cells per knee section were counted. Graphs represent mean ± SD (Data represents *n* = 7 mice per each group). *Significant differences between results in different group (i.e., *p* < 0.05).

HFD-mice is characterized by decreased intensity of safranin-O staining indicating proteoglycan degradation and increased Mankin scores mainly in tibia (Figures 2A,C). In DMM-operated mice, HFD administration led to more severe OA symptoms compared with DMM-operated CD mice (Figures 2B,C). IHC was performed to assess neoepitopes generated at the aggrecan cleavage sites produced by aggrecanase ADAMTS-4,5 (NITEGE) or matrix metalloproteinases (DIPEN) in murine articular cartilage. DIPEN and NITEGE are two commonly used matrix degradative markers to revaluate cartilage degeneration in OA (28, 29). As shown in Figure 2, HFD-mice showed an increased expression of DIPEN- and NITEGE-positive articular chondrocytes in knee joints compared to CD mice. DMM-operated mice fed with HFD also exhibited increase in production of the aggrecan neoepitopes, NITEGE and DIPEN compared with the DMM-treated CD mice (Figures 2A,D,E). Compared with HFD-mice, HFD~CD mice in sham group showed decreased Mankin score an indicative of OA cartilage pathology. However, the dietary switch from HFD~CD does not show any significant effect in the OA development induced by DMM surgery (Figures 2A–C). However, the upregulation of NITEGE and DIPEN was quantitatively reduced in HFD~CD mice compared to HFD group alone in OA DMM group (Figures 2B,D,E).

### Effects of Diet Switching on Synovial Pathology and Macrophage Expression in Mouse Model of OA

**Figure 3:**
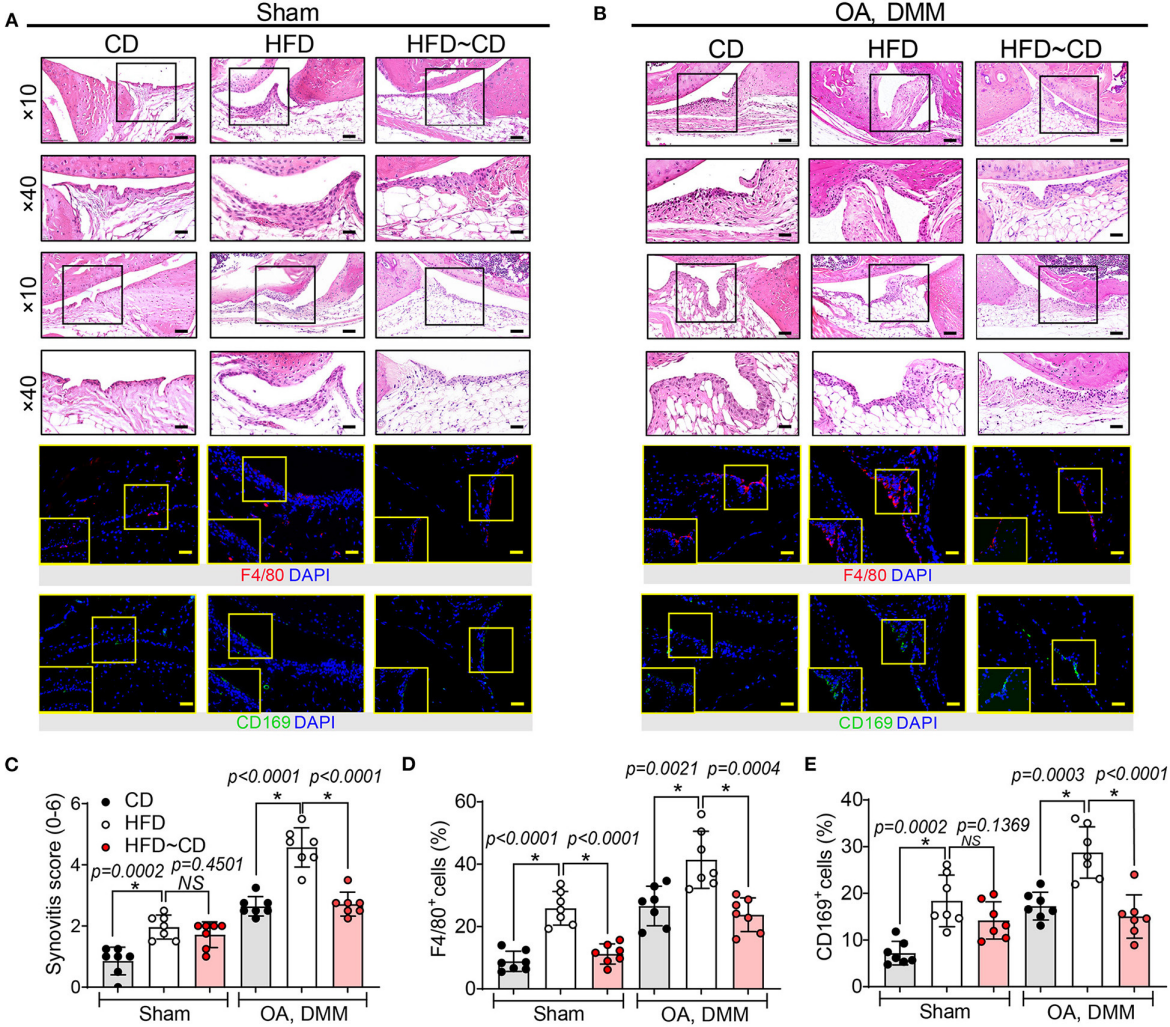
The presence of macrophage-associated synovitis in mouse model of osteoarthritis was reversible by diet intervention. **(A)** Top panel: Representative H&E stained sagittal sections of sham knee regions in mice fed a CD, HFD, or HFD~CD. Scale bars, 100 μm. The inset boxes in upper panels are shown at higher resolution in lower panels. Scale bars, 20 μm. Bottom panel: Similar sections were stained with F4/80 and CD169; the inset boxes are shown at higher resolution. Scale bars, 20 μm. **(B)** Top panel: Representative H&E stained sagittal sections of OA-operated knee regions in mice fed a CD, HFD or HFD~CD. Scale bars, 100 μm. The inset boxes in upper panels are shown at higher resolution in lower panels. Scale bars, 20 μm. Bottom panel: Similar sections were stained with F4/80 and CD169; the inset boxes are shown at higher resolution. Scale bars, 20 μm. *N* = 7 per group. **(C)** Synovial inflammation was assessed using synovitis scoring based on degree of cell thickness in the synovial lining layer and cell density of the synovial stroma. **(D,E)** The percentage of F4/80 **(D)** and CD169 **(E)**—positive cells per knee section were counted. Graphs represent mean ± SD (*N* = 7 per group). *Significant differences between results in different group (i.e., *p* < 0.05).

Next, we observed that mice fed HFD showed detectable synovial lining hyperplasia and an increment in fibrosis, and thus the synovitis score was higher than that observed in CD mice (Figures 3A,C). HFD administration also increased the number of F4/80+ macrophages in inflamed synovium, especially localized in the lining layer (Figures 3A,D). The OA-HFD group exhibited more severe synovitis correlated with extensive infiltration of F4/80-positive cell than in the OA-CD groups (Figures 3B,D). We examined whether HFD modulates the expression of inflammatory CD169+ macrophages in synovium. HFD mice manifested stronger immunoreactivity to CD169 in the synovium in comparison to CD mice (Figures 3A,E). The number of CD169+ macrophages further increased in the synovium in the OA-HFD group in comparison to the OA-CD group (Figures 3B,E). However, in the HFD~CD dietary switch group with OA, the population of F4/80^+^ macrophages in the synovium returned to similar levels as in CD group (Figures 3A,D). A clear diminution in the percentage of F4/80^+^ macrophages in the synovium of HFD~CD mice with OA surgery was observed in comparison to the OA-HFD group (Figures 3B,D).

### Weight Loss Leads to an Alteration in Systemic and Local Inflammation

**Figure 4:**
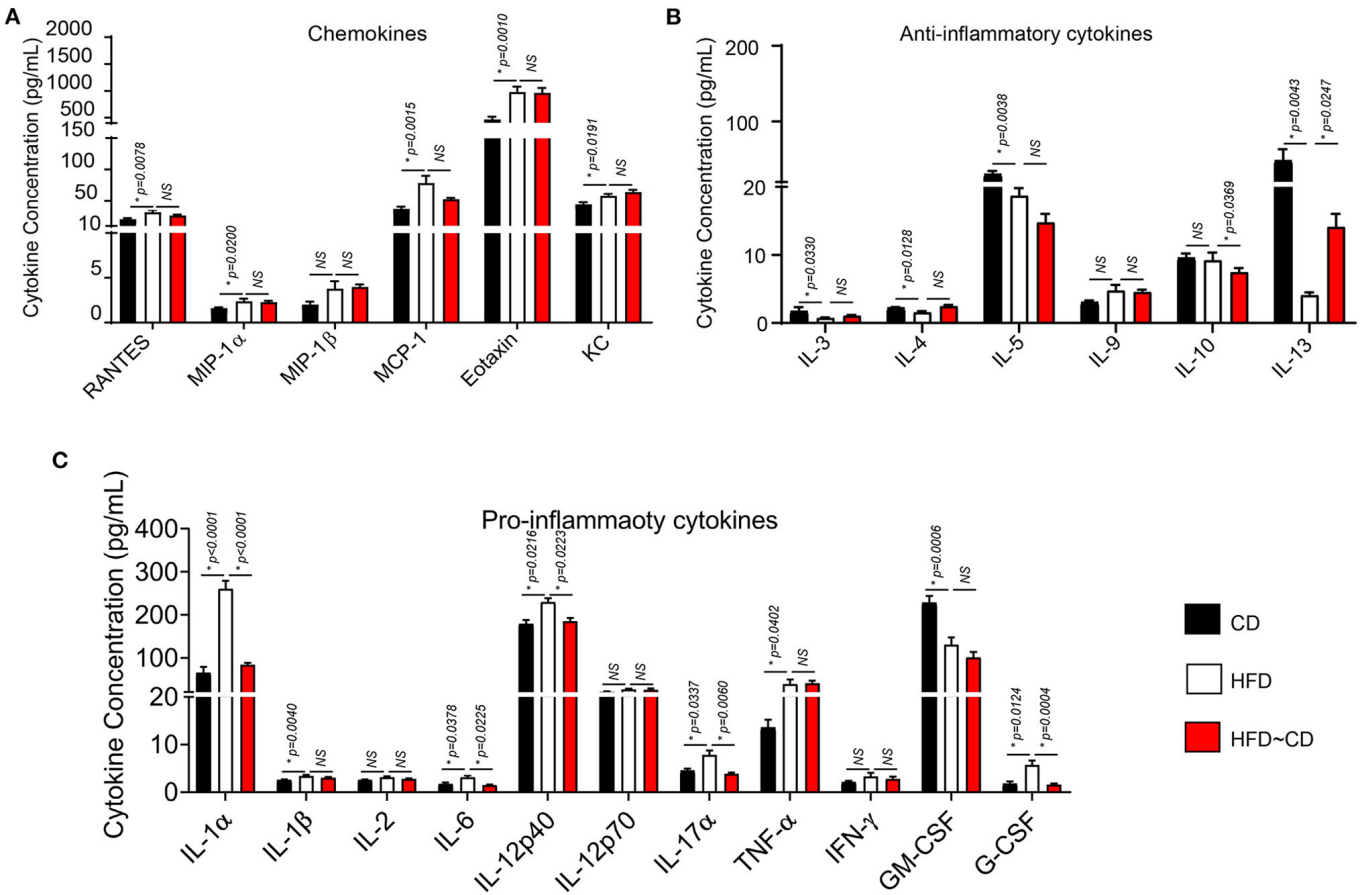
Effect of diet intervention-induced weight loss on systemic inflammation. **(A)** Measurement of serum chemokines in mice fed a CD, HFD, or HFD~CD. **(B)** Measurement of serum anti-inflammatory cytokines in mice fed a CD, HFD, or HFD~CD. **(C)** Measurement of serum pro-inflammatory cytokines in mice fed a CD, HFD, or HFD~CD. Graphs represent mean ± SD (*n* = 7 per group). *p* < 0.05 is considered significant.

We determined the serum inflammatory cytokine changes to test the effects of HFD followed by weight loss on the development of obesity-induced OA in mice. After 22 weeks of HFD, the chemokines RANTES, MIP-1α, MCP-1, EOTAXIN, and KC were increased in HFD subjects relative to CD controls while there were no differences in MIP-1β concentrations (Figure 4A). Moreover, HFD led to lower concentrations of anti-inflammatory cytokines including IL-3, IL4, IL-5, and IL-13 with no changes of IL-9 and IL-10 (Figure 4B). Additionally, concentrations of serum pro-inflammatory cytokines for IL-1α, IL-1β, IL-6, IL-12p40, IL-17α, and TNF-α were increased in HFD-mice, but there were no changes in IL-2, IL-12p70, G-CSFor IFN-γ (Figure 4C) compared with CD group. Notably, the concentration of GM-CSF in HFD-fed mice was markedly decreased (Figure 4C). Following dietary switch, HFD~CD mice had lower concentrations of pro-inflammatory cytokines IL-1α, IL-6, IL-12p40, and IL-17 compared to HFD-fed mice among the tested markers. Anti-inflammatory cytokines IL-4 and IL-13 were elevated, while circulating concentrations of IL-10 were decreased in the HFD~CD group compared with HFD-mice. Unlike alteration in cytokine expression, which changed in HFD~CD mice, there were no differences in chemokines that including MIP-1α, MIP-1β, RANTES, KC, and EOTAXIN (Figures 4A–C).

**Figure 5:**
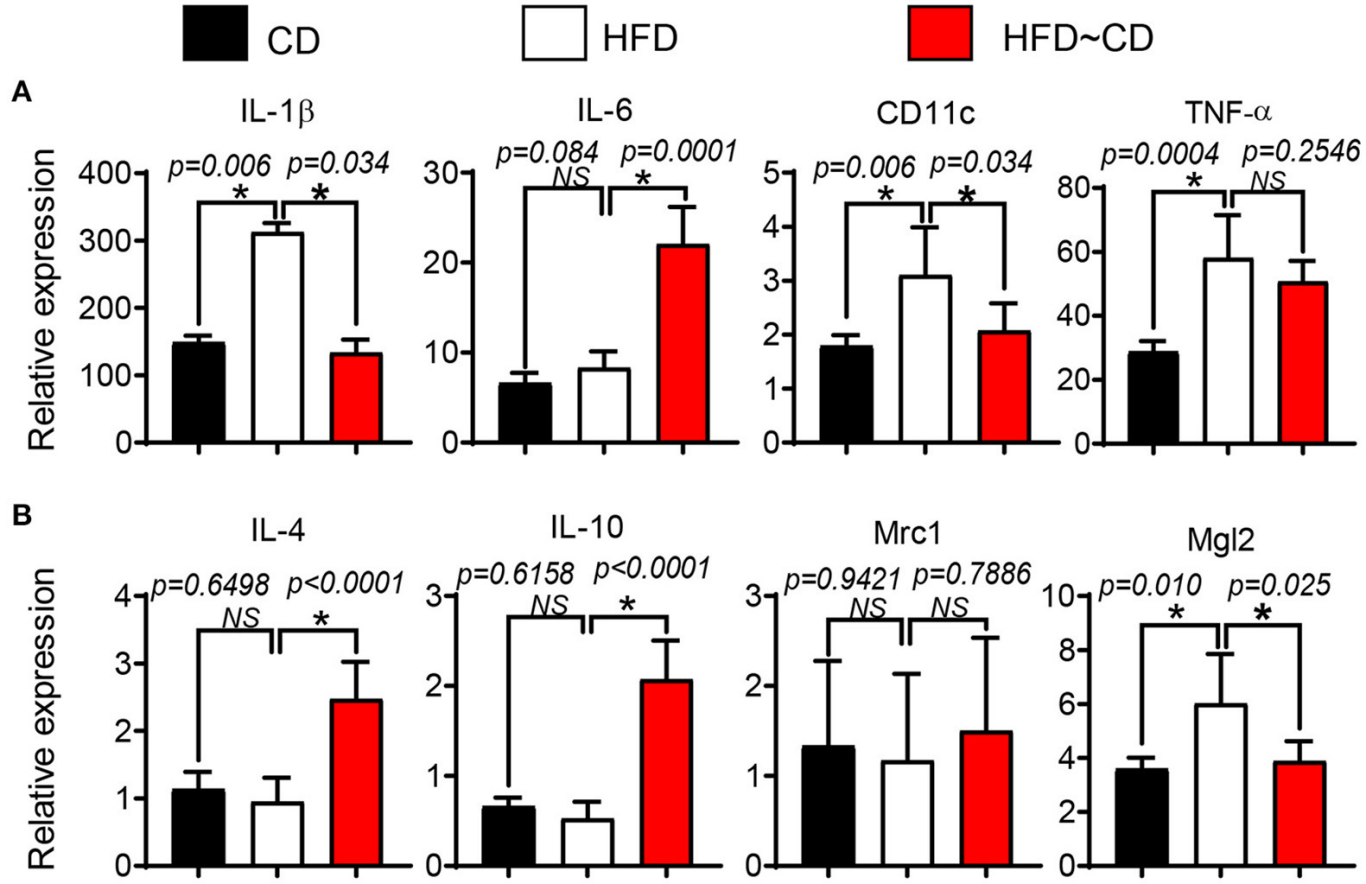
Effect of diet intervention-induced weight loss on local inflammation. **(A)** qPCR analysis of IL-1β, IL-6, CD11c, and TNF (pro-inflammatory markers) mRNA in synovial macrophages from CD-, HFD-, or HFD~CD- mice. **(B)** qPCR analysis of IL-4, IL-10, Mrc1, and Mgl2 (anti-inflammatory markers) mRNA in synovial macrophages from CD-, HFD-, or HFD~CD- mice. Data presented as mean ± SD of two independent experiments (each a pool of 3 mice) performed in triplicates. *p* < 0.05 is considered significant.

To test whether HFD diet induces pro-inflammatory conditions in the synovium, the gene expression in FACS-sorted CD45.2+F4/80+ macrophage-like synoviocytes was verified by qRT-PCR. Mice in the HFD group had a clear increase in pro-inflammatory markers including IL-1β, CD11c, and TNF in the synovium in comparison to controls (Figure 5A). However, anti-inflammatory markers such as IL-4, IL-10, Mrc1 were not affected by HFD in synovial macrophages (Figure 5B). We further examined the effect of diet intervention-induced weight loss on HFD-induced synovitis. Gene expression of pro-inflammatory markers IL-1β and CD11c were inhibited by diet reversal, but the expression of IL-6 mRNA in HFD~CD mice was increased compared with HFD-fed mice (Figure 5A). On the other hand, HFD~CD mice showed higher gene expression of anti-inflammatory cytokines IL-4 and IL-10. However, expression of Mgl2 was returned to normal levels in HFD~CD mice in comparison to HFD group (Figure 5B).

## Discussion

Diet and exercise are widely accepted as useful strategies for losing weight in obese adults (14). However, in obese patients with limited mobility and pain induced by OA, long-term maintenance of weight loss through exercise is challenging. Despite this limitation, weight loss has been recognized as an important approach to reduce OA symptoms in obese patients (13, 14). However, whether there are any benefits in joint tissue metabolism or structural progression is unknown. We determined whether weight loss induced by diet can effectively prevent OA development in rodent models of obesity and OA. We report 4 key findings: (1) obesogenic diets lead to OA changes in the knee joint in C57BL/6J mice; (2) obesogenic diets induce an accumulation of pro-inflammatory macrophages in the synovium and fat pad tissues; (3) replacement of the obesogenic diet with a control diet decreased systemic metabolic syndrome symptoms, circulating concentrations of inflammatory cytokines and inflammatory signaling activities; and (4) replacement of obesogenic diets with a control diet somewhat reduced synovial pro-inflammatory gene expression, and mitigated the effects of obesity-associated OA development.

Our results showed diet switching resulted in lowering of both body weight and serum inflammatory cytokine concentrations. Notably, we found that HFD~CD disrupted the clustering of cytokine expression which are mainly associated with increased adiposity (30, 31) such as IL-6 and IL-12. Our findings are consistent with the clinical findings that obese OA patients on a calorie-restricted diet without exercise showed weight loss and reduced plasma inflammatory biomarkers (32). Substantial evidence also supports that weight loss of 20% in patients with obesity and OA by gastric surgery led to an improvement in pain and physical function, and attenuation in systemic inflammation resulting in a structural improvement of cartilage (11). Moreover, systemic blockade of IL-6 improved cartilage structure in mouse OA models (33). Taken together, these results suggest that reduced dietary energy intakes reduce the systemic inflammatory status in obese rodents and humans. It is well-documented that the systemic production of inflammatory mediators contributes to cartilage degradation and activation of synoviocytes. Scanzello et al. has discussed that obesity-triggered low-grade inflammation consists of components such as inflammatory cytokines, abnormal metabolites that contribute to OA pathophysiology leading to cartilage matrix impairment, synovitis and subchondral bone remodeling (34). However, local synovial inflammation can be reflected outside the joint in the blood of patients with OA (35, 36). In this current study, we found that the dietary switch can modify some of the obesogenic diet-induced alterations in the immune response of synovium in mice along with improved systemic inflammatory status. These findings are in agreement with previous studies which showed that, in obese adults with knee OA, weight loss improved synovitis-induced pain (37). We have previously demonstrated that synovial macrophages in HFD-mice are predominantly polarized to the M1 pro-inflammatory phenotype, while M2 activated macrophages are also present (15, 16). Therefore, we hypothesized that improvement of systemic inflammation by dietary switch reduced obesity-associated synovitis by reversing the inflammatory status in the synovium. The effect of dietary switch on synovial macrophage phenotype in obesity-related OA remains unknown, although the cytokine presence in the OA synovial macrophages has been extensively studied and a close relationship between cytokine expression and progression of OA was shown in previous studies (15, 38).

It is interesting to note that there is no differences between HFD (DMM) and HFD~CD (DMM) on cartilage destruction assessment. However, further longitudinal investigations in future could shed light on the influence of diet control on the onset and development of OA at various time points. Although we did not observe significant differences between these two groups on cartilage destruction assessment, macrophage-associated synovitis was significantly improved after diet intervention along with decreased systemic inflammatory conditions in the animals. Our previous study has shown that an increase of synovial inflammation halfway through the 16-week-diet (i.e., week 8), when the cartilage still appeared normal (39). Larranga-Vera et al. also suggested that the aggravation in synovial inflammation induced by HFD is not a secondary event resulting from a pathological change in the cartilage (40). This indicated the immune response in the synovium preceded cartilage degeneration which became more apparent at the experimental endpoint. As such, therapeutic strategies targeting the inflammatory synovium can be pivotal to attenuate the severity of OA, but also prevent the onset of disease. In this study, we observed that weight loss with HFD~CD dietary switch group resulted in change of synovial macrophages from a pro-inflammatory phenotype with high expression of CD11c to an anti-inflammatory or resolving state with a reduction in CD11c expression. Considering the pro-inflammatory properties of saturated fatty acids in obese individuals (38), this suggests that the low-fat content in the diet might play an important role in the conversion of synovial macrophage phenotype and resolution of synovial inflammation in HFD~CD-induced weight loss.

Our results show that mice on HFD~CD increased concentrations of the pro-inflammatory cytokine IL-6. Although IL-6 is commonly known as a pro-inflammatory cytokine, it is a multifunctional cytokine with anti-inflammatory activity as it increases the production of anti-inflammatory mediators IL-10 and IL-4 by the suppression of IFNγ signaling (41–43). Mgl2 mRNA encoding the CD301b protein is highly expressed in the M2-like macrophage. In this study, we observed that the obese mice on a HFD~CD exhibited decreased expression of Mgl2 mRNA in macrophage-like synoviocytes, consistent with the reduced expression of Mgl2 after weight loss in obese patients (44). The decreased Mgl2 expression in synovial macrophages sorted from HFD~CD mice might be due to its important roles in maintaining positive energy balance and glucose metabolism. HFD-fed Mgl2-DTR mice treated with diphtheria toxin showed weight loss, enhanced insulin sensitivity and gluconeogenesis, accompanied by a marked reduction in circulation of RELM α, a multifunctional cytokine produced by mononuclear phagocytes with roles in promoting insulin resistance and obesity (45). Taken together, these results suggest that the beneficial effect of HFD~CD on knee joint may be due to the upregulation of IL-6 and downregulation of Mgl2 in the synovial macrophage. Thus, HFD~CD diet switching improves systemic metabolism, modifies inflammatory status and supports the important association between obesogenic diet-induced obesity (DIO) and the development of OA. However, an overall profile of cytokines, chemokines and other signaling proteins of the synovium from the two rodent groups is required to clarify the effect of dietary switch in local inflammation of obesogenic diet-induced obesity-associated OA.

The application of dietary intervention and protein augments as a therapeutic strategy on obesity-induced diseases are widely reported. The obesity-associated hyperinsulinemia and liver dysfunction are reversible by diet-mediated weight loss in DIO mouse model (46). Further investigation on these non-pharmacological interventions could shed light on the influence of diet on practical pathological changes.

## Conclusion

In conclusion, our data demonstrate that obesogenic diets induce systemic and synovial inflammation. Further long-term studies are required to assess if weight loss through dietary switching is an intervention that remodels the systemic and local inflammatory status and slows down the progression of OA cartilage degeneration.

## Data Availability Statement

The original contributions generated for the study are included in the article/supplementary material, further inquiries can be directed to the corresponding authors.

## Ethics Statement

The animal study was reviewed and approved by Institutional Animal Care and Use Committees of Central-South University (CSU), China.

## Author Contributions

AS, XW, RC, YX, XM, and IP conceived the study conception and design. XW, HL, LM, YL, and XM performed the animal experiments. AS, XW, YX, and IP conducted the histology and/or analyzed the data. RC provided advice on osteoarthritis experiments. All authors have read and approved the final manuscript.

## Conflict of Interest

The authors declare that the research was conducted in the absence of any commercial or financial relationships that could be construed as a potential conflict of interest.
